# Characterization of drug binding within the HCN1 channel pore

**DOI:** 10.1038/s41598-018-37116-2

**Published:** 2019-01-24

**Authors:** Jérémie Tanguay, Karen M. Callahan, Nazzareno D’Avanzo

**Affiliations:** 1 Department of Physics, Université de Montréal, Montréal, Canada; 2 Department of Pharmacology and Physiology, Université de Montréal, Montréal, Canada

## Abstract

Hyperpolarization-activated cyclic nucleotide-gated (HCN) channels mediate rhythmic electrical activity of cardiac pacemaker cells, and in neurons play important roles in setting resting membrane potentials, dendritic integration, neuronal pacemaking, and establishing action potential threshold. Block of HCN channels slows the heart rate and is currently used to treat angina. However, HCN block also provides a promising approach to the treatment of neuronal disorders including epilepsy and neuropathic pain. While several molecules that block HCN channels have been identified, including clonidine and its derivative alinidine, lidocaine, mepivacaine, bupivacaine, ZD7288, ivabradine, zatebradine, and cilobradine, their low affinity and lack of specificity prevents wide-spread use. Different studies suggest that the binding sites of these inhibitors are located in the inner vestibule of HCN channels, but the molecular details of their binding remain unknown. We used computational docking experiments to assess the binding sites and mode of binding of these inhibitors against the recently solved atomic structure of human HCN1 channels, and a homology model of the open pore derived from a closely related CNG channel. We identify a possible hydrophobic groove in the pore cavity that plays an important role in conformationally restricting the location and orientation of drugs bound to the inner vestibule. Our results also help explain the molecular basis of the low-affinity binding of these inhibitors, paving the way for the development of higher affinity molecules.

## Introduction

Hyperpolarization-activated cyclic-nucleotide gated (HCN) channels are the molecular correlate of the currents I_f_ or I_h_ in sinoatrial node (SAN) cells and neurons. Four mammalian isoforms have been identified (HCN1-4) with 60% sequence identity among them. Topologically, HCN channels resemble voltage-gated potassium (Kv) channels, however, functionally they are spectacularly different. HCN channels are formed by homo- or hetero-tetrameric assembly of subunits^1^. Each subunit contains 6 transmembrane α-helices (S1–S6), a re-entrant loop between the S5 and S6 helices that forms the selectivity filter and a C-terminal cyclic-nucleotide binding domain (CNBD) attached to the S6 via an 80 amino acid C-linker. Like other voltage-gated channels, HCN channels contain a positively charged S4 helix that functions as a voltage sensor that moves with the same directionality as voltage sensors of other channels^2,3^. However, HCN channels slowly activate at very negative (hyperpolarized) membrane potentials in which other voltage-gated cation channels close. Electrophysiological recordings have characteristic properties, including activation upon membrane hyperpolarization, a lack of voltage-dependent inactivation, conduction of Na^+^ and K^+^, a shift in the activation curve due to direct interaction with cAMP and cGMP, and inhibition by external Cs^+^^4^. The rates of opening and closing differ for each mammalian HCN isoform. HCN1 channels activate in less than 300 ms, while HCN4 channels require seconds to open. Moreover, the half-maximal voltage for activation (V_1/2_) for HCN1 and HCN3 are significantly depolarized compared to HCN2 and HCN4. HCN isoforms also differ from one another in their response to cyclic nucleotides. cAMP shifts the V_1/2_ in HCN2 and HCN4 by +15 mV, while HCN1 and HCN3 are only weakly modulated, with cAMP inducing shifts in V_1/2_ of less than +5mV^5–8^.

HCN1 and HCN2 channels are widely expressed in the central and peripheral nervous systems where they are open at sub-threshold potentials and play roles in setting resting membrane potentials, dendritic integration, neuronal pacemaking, and establishing action potential threshold. HCN1 knockout mice have impaired motor learning^9,10^ and enhance susceptibility to seizures^11^. HCN2 knockout mice present symptoms of absence epilepsy and tremoring^12^, and do not demonstrate neuropathic pain in response to mechanical or thermal stimuli^13^. The gain of function and loss of function mutations in HCN1 and 2 are linked to various genetic epilepsies in humans^14–18^. Altered HCN-cAMP signaling in prefrontal cortex networks also appears to contribute to the working memory deficits in schizophrenia and stress^19–21^. Mutations in the scaffolding protein SHANK3 may predispose people to autism by inducing an I_h_ channelopathy with increased neuronal input resistance, enhanced neuronal excitability and reduced synaptic transmission^22^. Additionally, HCN4 is the principal component of I_h_ in all mammalian sinoatrial node (SAN) and other cardiac conduction tissue^5,23–26^. HCN4^−/−^ resulted in embryonic death in mice due to a failure to generate mature pacemaking cells^12,27^ while HCN4 conditionally deficient mice have a 70–80% reduction in SAN I_h_^28^. Genetic variants in HCN channels have been linked to cardiac disorders including sinus node dysfunction, atrial fibrillation^29–39^, ventricular tachycardia^40–42^, atrio-ventricular block^43^, Brugada syndrome^38,44^, sudden infant death syndrome^45,46^, and sudden unexpected death in epilepsy^47^.

Since neuronal HCN channels are open at sub-threshold potentials, and make the cell membrane less responsive to incoming inputs, they are excellent targets for fine-tuning of intrinsic neuronal excitability. Inhibition of cardiac I_h_ by bradycardic agents such as ivabradine have been found useful in reducing the incidence of cardiovascular mortality and hospitalisation for some subclasses of heart failure^48^. Moreover, since HCN channel expression is largely limited to the heart and nervous system, and HCNs are not found in vascular tissue, targeted inhibition of HCN channels has strong therapeutic potential for several cardiac and neuronal disorders, without inducing any adverse effects on pulmonary and vascular smooth muscle tone. While several molecules that target HCN channels have been identified, including ZD7288^49–53^, zatebradine^54,55^, cilobradine^54,55^, and ivabradine^56–58^, their low affinity and lack of isoform specificity prevents wide-spread use of these current HCN inhibitors. Additionally, HCN channels have been shown to interact with molecules such as clonidine^59^ and its derivative alinidine^60^, bupivacaine, lidocaine, and mepivacaine^61^. However, these molecules are not HCN specific and interact with numerous other channels and receptors as well.

In light of the recent high-resolution structures of human HCN1^62^ and the related eukaryotic CNG channel^63^ we set out to characterize the interactions of 9 known HCN inhibitors within the pore using a computational docking approach. Our data provide insights into the drug binding modes and potentially explain the molecular bases for their low affinities.

## Results

### Docking to the closed HCN1 pore

**Figure 1:**
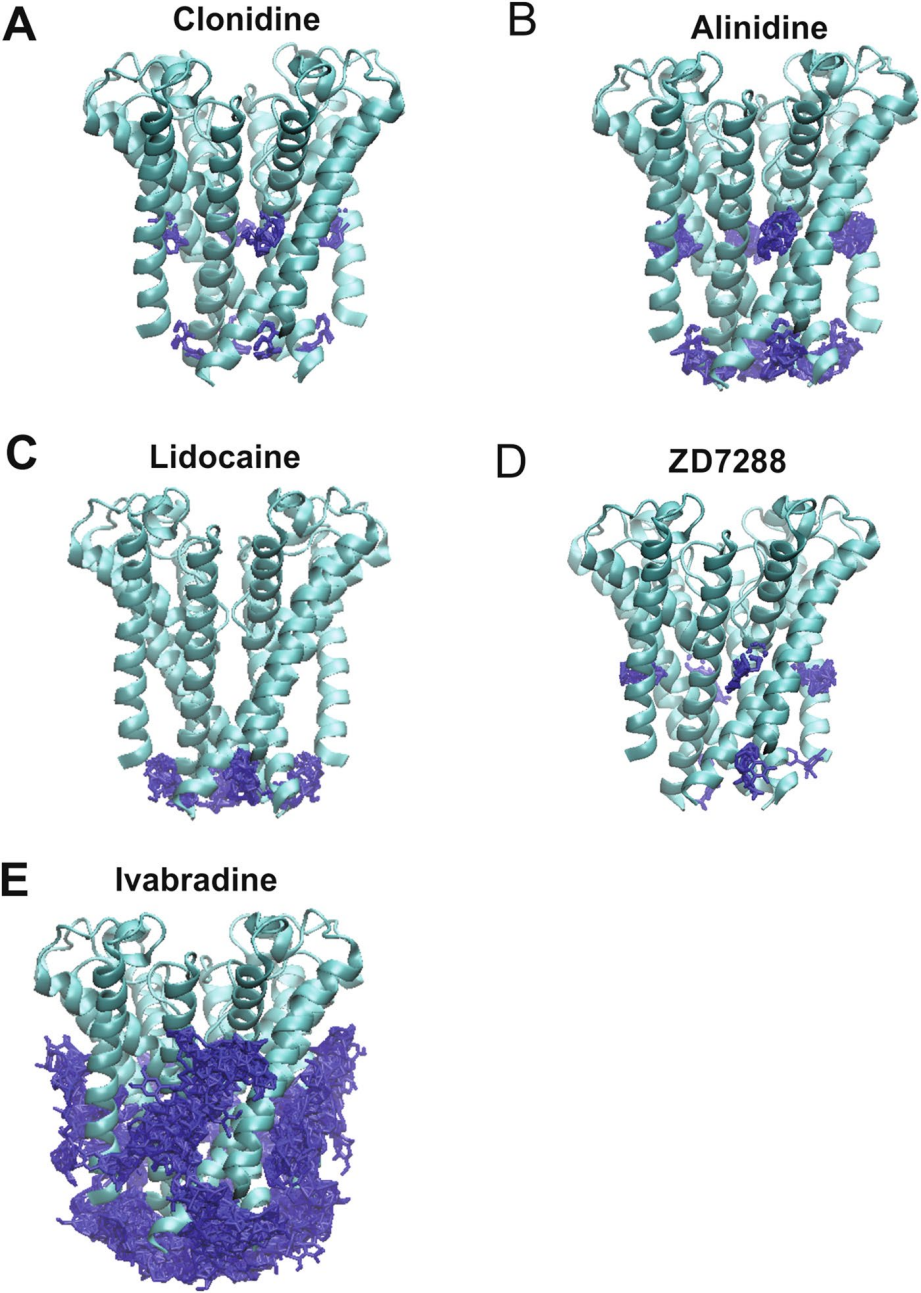
Docking of HCN blockers to the apo-closed HCN1 pore. The results from 500 attempts to dock clonidine (**A**) alinidine (**B**) lidocaine (**C**) ZD7288 (**D**) and ivabradine (**E**) to the closed pore of the cryo-EM structure (PDB: 5U6O). Despite these ligands being known to block or be “trapped” in the closed state, none of the 500 docked poses for any of these inhibitors were observed in the pore cavity. These data indicate that the apo-closed pore conformation must differ from the ligand-trapped closed pore.

500 docking attempts were made for each ligand to the closed human HCN1 pore extracted from the cryo-EM structure (PDB: 5U6O)^62^. For all ligands, 0 of the 500 docking conformations resided in the central cavity (Fig. 1). Similarly, ligands were not observed to bind within the pore cavity in any of the 500 docking attempts to the cAMP-bound HCN1 pore (PDB: 5U6P) (Supp. Fig. 3). We estimate the diameter of the central cavity measured at Y386 of opposite subunits to be 7.7 Å, enabling little more than 2–5 waters to occupy this space. Given that ligands such as ivabradine and ZD7288 are much larger and have been suggested to be trapped in the closed state^56–58,64,65^, our data suggests that the ligand-trapped closed state must differ from the apo-closed state that was observed in the cryo-EM structures. We therefore generated an open state model of human HCN1, based on the structure of the closely related eukaryotic CNG channel TAX-4^63^.

### Clonidine and alinidine binding to the open HCN1 pore

**Figure 2:**
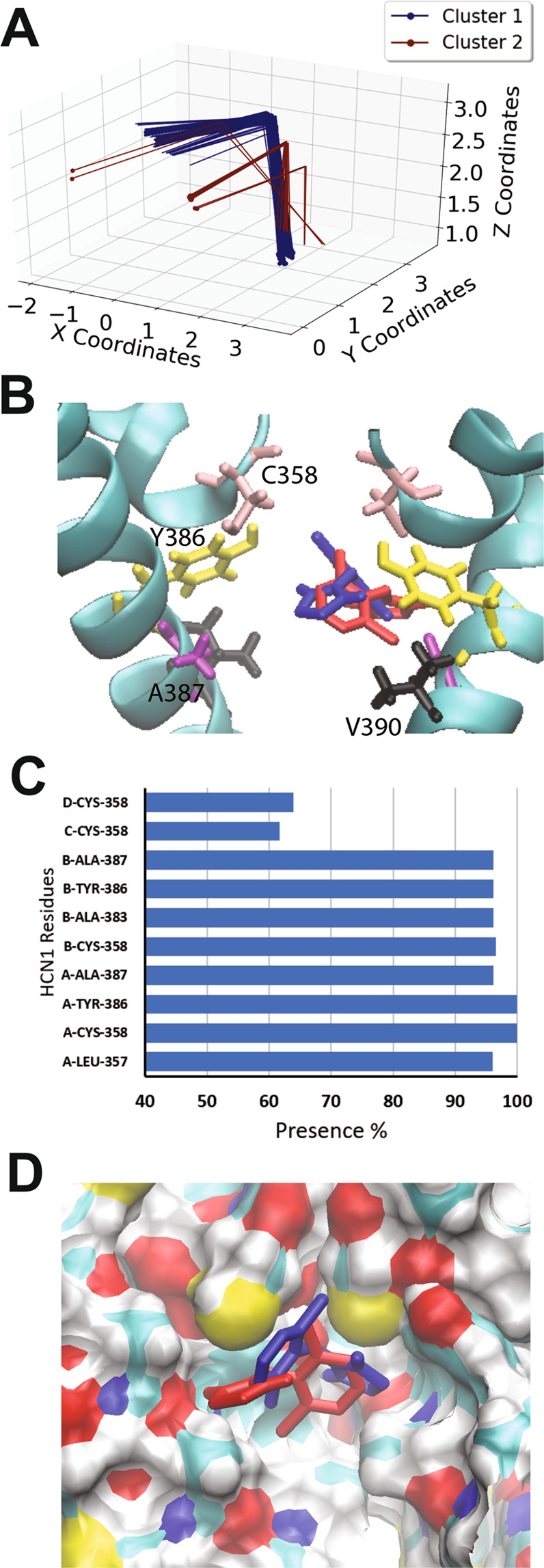
Docking of clonidine to the open HCN1 pore. (**A**) Clustering analysis of the 500 docking attempts. Docks can be placed into 2 clusters according to our automated algorithm. (**B**) Representative poses from cluster 1 (95% of poses; blue) and cluster 2 (5% of poses; red) are shown. Residues C358 (pink), Y386 (yellow), A387 (purple) and V390 (black) are highlighted. (**C**) The frequency of residues within 4 Å of the ligand for each pose was assessed and is indicated as a percentage. (**D**) A comparison of clonidine poses in cluster 1 (blue) and cluster 2 (red). The ligand fits into a hydrophobic groove lined by residues C358, Y386, and A387.

Clonidine, a well-known α2-adrenergic receptor agonist, also inhibits HCN2 and HCN4 with IC50’s of ~3–10 µM, shifting their voltage dependence to more hyperpolarized potentials by 10–20 mV, and is 4–10 fold less potent in HCN1^59^. Docking of clonidine to the open HCN1 pore lead to the identification of two putative clusters within the pore cavity that cannot be distinguished based on binding energy alone. The autodock algorithm estimates a binding energy per non-hydrogen atom of −0.357 +/− 0.01 kcal/mol. Both of these clusters are well defined by position and orientation, with RMSDs of 0.02 Å and 0.17 Å from the reference pose within each clusters. The first cluster (Fig. 2A blue) contained 95% of the docked poses, and fit like a “lock and key” into a groove in the internal cavity formed by residues C358, Y386, and A387. In this conformation, a persistent hydrogen bond could be observed between the imidazole hydrogen of clonidine, and the hydroxyl oxygen of Y386. Another stabilizing hydrogen bond is observed between the N1 nitrogen and the hydroxyl hydrogen of the same Y386. In the second cluster, containing only 5% of the poses (Fig. 2B red), the clonidine is stabilized a single hydrogen bond between the imidazole hydrogen and the backbone carbonyl oxygen of A383 and reciprocating anion- π interactions between clonidine and the Y386 aromatic rings.

**Figure 3:**
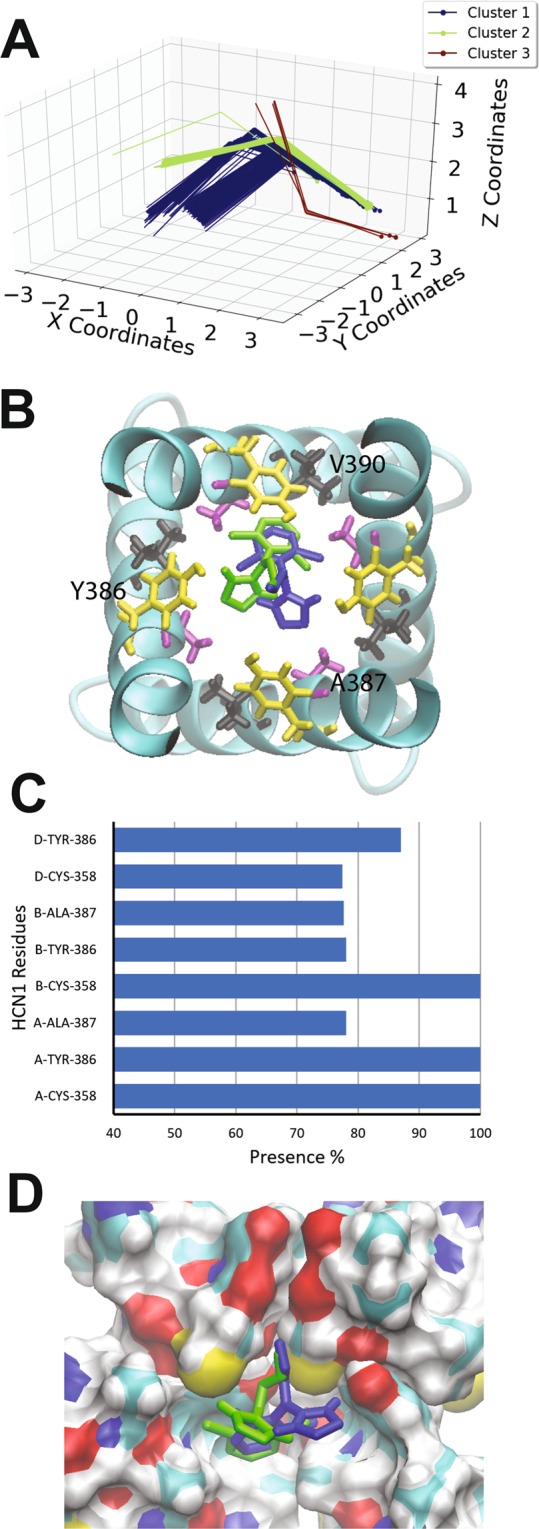
Docking of alinidine to the open HCN1 pore. (**A**) Clustering analysis of the 500 docking attempts. Docks can be placed into 3 clusters according to our automated algorithm. Cluster 1 (blue), cluster 2 (green) and cluster 3 (red) contained 319, 177 and 4 poses respectively. (**B**) Top view of the HCN1 pore with representative poses from cluster 1 (blue) and cluster 2 (green) are shown with residues C358 (pink), Y386 (yellow), A387 (purple) and V390 (black) highlighted (**C**) The frequency of residues within 4 Å of the ligand for each pose was assessed and is indicated as a percentage. (**D**) A comparison of clonidine poses in cluster 1 (blue) and cluster 2 (green). The ligand fits into a hydrophobic groove lined by residues C358, Y386, and A387.

Alinidine (*N*-allyl-clonidine) is a clonidine derivative with differing pharmacological effects, including its bradycardic^66–68^ and analgesic properties^69^. Alinidine induces the slowing of spontaneous activity of rabbit SAN accompanied by a 10% prolongation of the action potential duration^60^ and by reducing the steepness of diastolic depolarization of Purkinje fibres with limited side effects on action potential duration and inotropic state^70^. Alinidine shifts the voltage-dependence of I_h_ activation to more negative voltages and reduced maximal conductance by 27% with no use or frequency dependence, indicating that alinidine binds equally well to HCN open and closed channel states. Alindine docking to the human HCN1 open pore resulted in 3 clusters (Fig. 3A), however, the third cluster (red) was populated by only 4 poses and bound with higher energy. Common to each of the two highest occupied and lowest energy clusters, the α-butylene group of alinidine extends into the S4 ion binding site of the selectivity filter, and would be expected to displace an ion in this site (Fig. 3C). Similar to clonidine, in these two clusters, alinidine fit like a “lock and key” into a groove in the internal cavity formed by residues C358, Y386, and A387. The binding energy per non-hydrogen atom was estimated to be −0.320 +/− 0.001 kcal/mol, −0.332 +/− 0.001 kcal/mol for clusters 1 and 2 respectively, which are indistinguishable according to Autodock. 319 docked poses (64%) reside in cluster 1, where a persistent hydrogen bond between the imidazole hydrogen of clonidine, and the hydroxyl oxygen of Y386 is observed. In addition, in this cluster, alinidine is stabilized by an anion-π or parallel-displaced π-stacking interaction between its aromatic group and the Y386 aromatic ring of another subunit. Poses in cluster 2 (177 of 500 docks) bind in the similar region with a different orientation, such that hydrogen bonding between the imidazole ring and the Y386 hydroxyl groups can still occur, but only parallel-displaced π-stacking between the alinidine and neighbouring Y386 aromatic rings can occur, and not an anion-π interaction.

The overall examination of clonidine and alinidine binding to the HCN1 pore indicate that a hydrophobic groove generated by residues C358, Y386, and A387 can assist in conformationally restraining the ligand. Moreover, Y386 of neighbouring subunits co-ordinate these ligands through a combination of hydrogen bonding and π-bonding interactions.

### Binding of lidocaine to the open HCN1 pore

**Figure 4:**
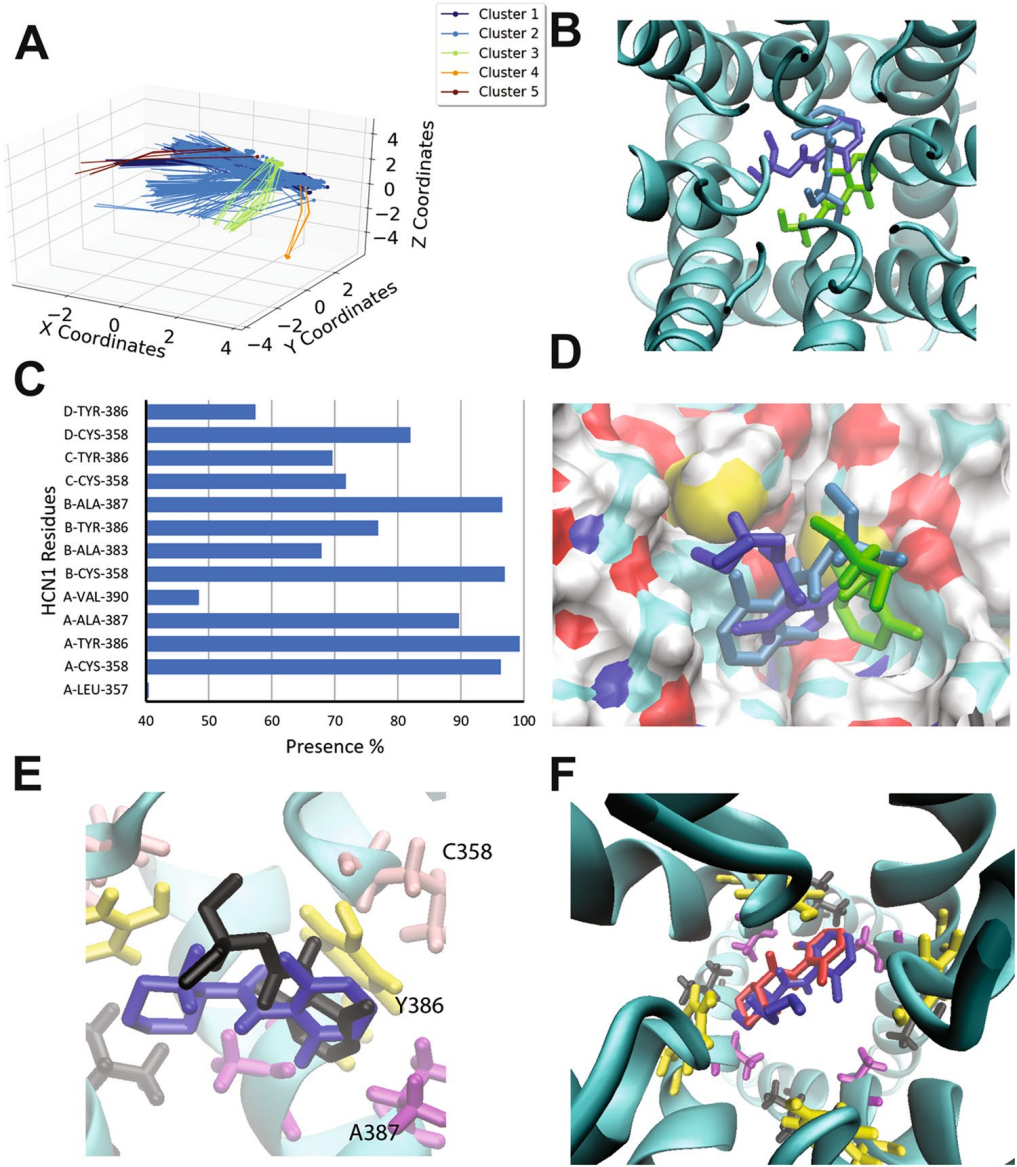
Docking of lidocaine and derivatives to the open HCN1 pore. (**A**) Clustering analysis of the 500 docking attempts. Docks can be placed into 5 clusters according to our automated algorithm. (**B**) Top view of the HCN1 pore with representative poses from cluster 1 (blue) cluster 2 (cyan) and cluster 3 (green) are shown (**C**) The frequency of residues within 4 Å of the ligand for each pose was assessed and is indicated as a percentage. (**D**) Side view of representative poses from cluster 1 (blue) cluster 2 (cyan) and cluster 3 (green) are shown. The ligand aromatic ring of lidocaine fits into a hydrophobic groove lined by residues C358, Y386, and A387, while the amine portion of the ligand points toward the central pore axis. (**E**) Overlay of lidocaine (black) and mepivacaine (blue) docked in the pore of HCN1 channels. Mepivacaine docks with both rings in hydrophobic groove formed by neighbouring subunits, while lidocaine’s amine points toward the pore axis. (**F**) Mepivacaine (red) and bupivacaine (blue) dock similarly, however, the butyl group on the aromatic ring extends toward the central axis of the pore cavity likely enhancing the occlusion of ions.

Lidocaine, mepivacaine and bupivacaine are local anesthetics known to inhibit voltage-gated sodium (Nav) channels^71^. Recently, these molecules have been shown to also inhibit HCN currents in DRG neurons with similar potencies^61^, suggesting clinical relevance. Since HCN blockade by lidocaine appears to occur from the inside of the cell^72^, we performed docking experiments of lidocaine to our model of the open human HCN1 pore. 5 clusters of docking poses were identified, with the binding sites lined by residues L357, C358, A383, Y386, A387, and V390 (Fig. 4). Similar to what we observed for clonindine and alinidine, the most occupied cluster and with low energy (cluster 2; occupied by 306 poses), is stabilized by π-stacking interactions of the aromatic rings of the ligand and Y386. This enables the ligand to sit deeper in the hydrophobic groove than clonidine and alinidine (Fig. 4D) and close enough to interact with V390. A hydrogen bond network is also formed between the amide hydrogen of lidocaine and the hydroxyl oxygen of the same Y386 that π-stacks with the ligand, as well as the sulfhydryl sulfur atom of C358. Cluster 1 (occupied by 136 poses) is rotated such that the aromatic ring can no longer π-stack, and the amide bond is rotated such that the hydrogen bond network is now formed by the oxygen of the amide bond in lidocaine, and the hydrogen atoms of the Y386 and C358 sidechains. In general, the aromatic of ring in most lidocaine poses is constrained in the hydrophobic groove, independent of whether or not π-stacking can occur, while the amine portion points toward the central pore axis. Notably, the binding energies per non-hydrogen atom are comparable to clonidine and alinidine, with a mean of −0.307 kcal/mol and a maximum of −0.333 kcal/mol. The highest energy poses occupied a cluster (cluster 4; orange) with only 4 out of 500 poses with the ligand displaced against one of the S6 pore helices and may represent a local minimum, and not the physiologically relevant binding pose.

Bupivacaine and mepivacaine are lidocaine derivatives with ringed R-groups replacing the ethylamine. Bupivacaine and mepivacaine had comparable binding energies per non-hydrogen atom to lidocaine (−0.320 kcal/mol and −0.332 kcal/mol respectively). However, we found mepivacaine to dock with greater complementarity into the pore grove than lidocaine. Poses can be arranged into 2 clusters (Supp. Fig. 3) with the ligands simply rotated by approximately 180° around the center of the molecule. Notably, while the aromatic ring of lidocaine resides in the hydrophobic groove, its amine does not arrange itself into the portion of the hydrophobic groove formed by the neighboring subunit, but rather points toward the central pore axis (Fig. 4E). On the other hand, both ring structures of mepivacaine can arrange themselves into both portions of the hydrophobic groove formed by neighbouring subunits. Interestingly, bupivacaine largely appears to dock into the same positions as mepivacaine, with the butyl group extended toward the central axis of the pore cavity (Fig. 4F), similar to the amine group of lidocaine. Considering that lidocaine, bupivacaine and mepivacine have increasing IC50’s^73^, our results suggest that while the nitrogen containing ring is entropically favourable to help orient the “caines” into the hydrophobic groove, there may be an important role in occluding permeant ions by having a greater portion of the ligand occupying the central axis of the pore cavity.

### ZD7288 binding to the open HCN1 pore

**Figure 5:**
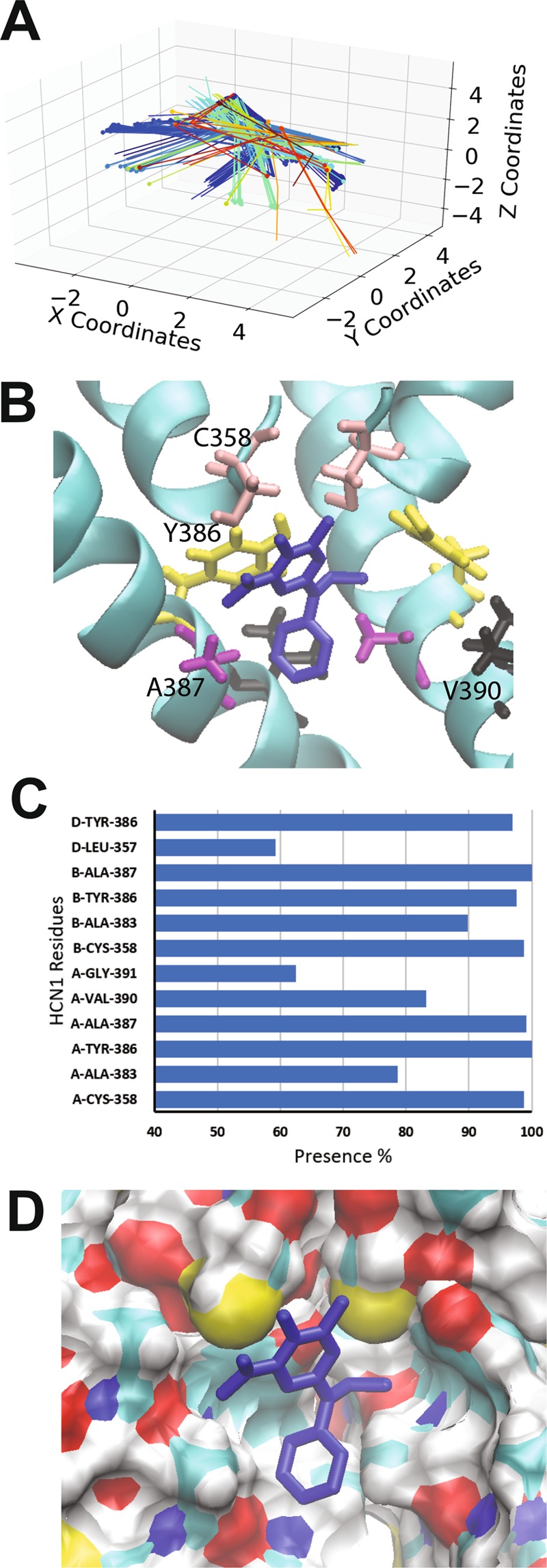
Docking of ZD7288 to the open HCN1 pore. (**A**) Clustering analysis of the 500 docking attempts. Docks can be placed into 24 clusters according to our automated algorithm, indicating that ZD7288 does not bind in a preferred orientation. (**B**) A representative pose of ZD7288 with residues C358 (pink), Y386 (yellow), A387 (purple) and V390 (black) highlighted. (**C**) The frequency of residues within 4 Å of the ligand for each pose was assessed and is indicated as a percentage. (**D**) ZD7288 does not fit nicely into the hydrophobic groove and the aromatic sidechain of Y386 does not appear to contribute to the mechanism of binding as it does for other ligands.

ZD7288 is an open-state blocker of HCN channels^51,65,74,75^ with bradycardic and antianginal activity in animal models^49,76^. ZD7288 induces at ~15 mV hyperpolarizing shift in voltage-dependent I_h_ activation and reduces maximal activity by more than 50%^49^. This drug also reduces the generation of hippocampal epileptic discharges in rabbits^77^ and reverse pain behavior and spontaneous discharges in injured rat nerve fibers^78–80^. The 500 docking attempts for ZD7288 against the human HCN1 open pore resulted in 24 clusters (Fig. 5A), with 271 poses (54%) in one cluster, interacting with residues L357, C358, A383, Y386, A387, V390 and G381 of 3 subunits within the tetramer (Fig. 5B,C). Poses in this cluster are largely stabilized by hydrogen bonds between the ligand’s nitrogen atoms and the hydroxyl groups of Y386. The remaining clusters reside on the same plane, and interact with the same residues, however, are not rotationally constrained, thereby reducing the number of hydrogen bonds formed. However, the binding energies per non-hydrogen atom between the best occupied cluster (cluster 1; −0.305 +/− 0.003 kcal/mol) and the other clusters (all ranging between −0.29 and −0.31) are indistinguishable. This suggests that binding of ZD7288 in HCN channels does not likely occur with a preferred orientation, and that the ligand is relatively free to rotate within the pore cavity. Moreover, unlike the binding of clonidine, alinidine and lidocaine, π-stacking interactions between the aromatic portions of the ligand, and the aromatic sidechain of Y386 do not appear to contribute to the mechanism of binding of ZD7288.

### Ivabradine binding to the open HCN1 pore

**Figure 6:**
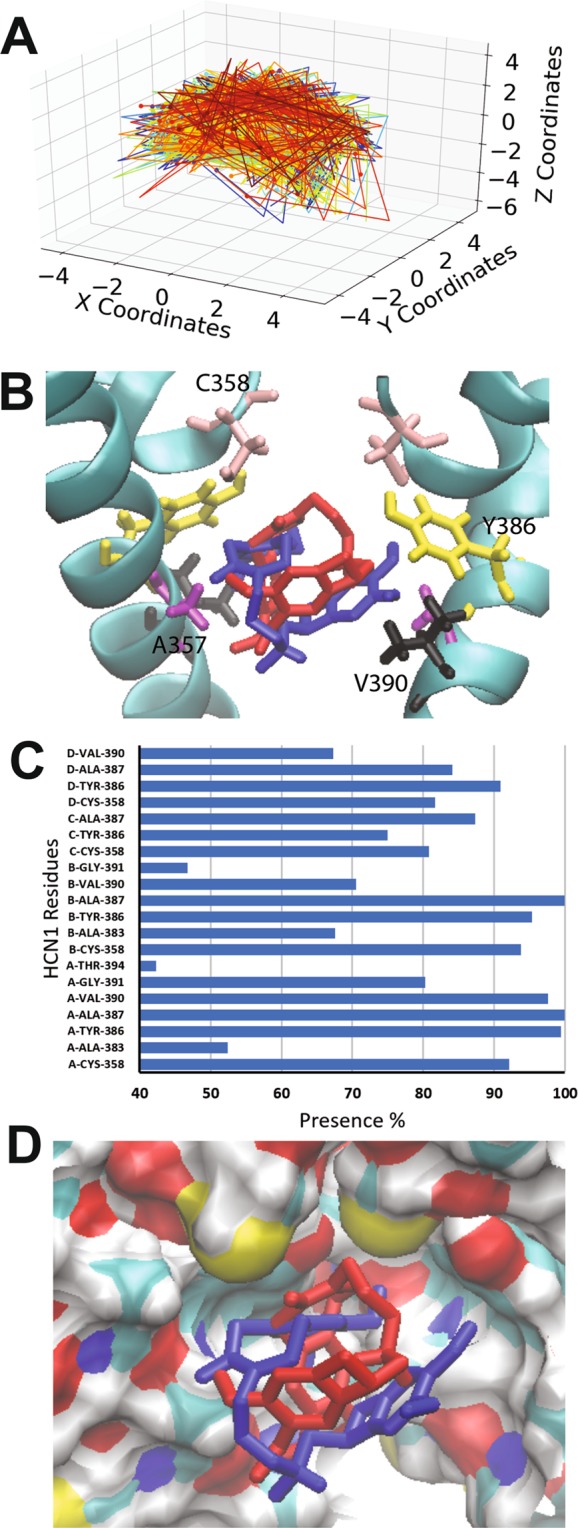
Docking of ivabradine to the open HCN1 pore. (**A**) Clustering analysis of the 500 docking attempts. Docks cannot be clustered according to our automated algorithm, indicating that ivabradine does not bind in a preferred orientation. (**B**) Two poses of ivabradine are shown with residues C358 (pink), Y386 (yellow), A387 (purple) and V390 (black) highlighted. Ivabradine in all poses folds into a U-shape, however, is free to rotate 360° in the pore cavity. (**C**) The frequency of residues within 4 Å of the ligand for each pose was assessed and is indicated as a percentage. (**D**) None of the ivabradine fit nicely into the hydrophobic groove which is largely prevented by the presence of the methoyx groups on the aromatic ring.

Ivabradine was the first clinically approved molecule that specifically targeted HCN channels for the treatment of heart failure^81–83^. Ivabradine block of HCN4 can occur only from the intracellular side^58^ when the channels are opened by hyperpolarization with enhanced binding upon frequent changes in the direction of ion flow^56–58^. In contrast, HCN1 channels can also be inhibited from the closed state^58^. Intriguingly, the 500 docking attempts of ivabradine to the human HCN1 open pore could not be clustered using our algorithm. The ligand binds in the pore cavity in a U-shaped configuration (Fig. 6B), without positional or orientational restriction (Fig. 6A). Consequently, ivabradine can interact with residues C358, A383, Y386, A387, V390, G391, and T394 of all 4 subunits. The binding energy per non-hydrogen atom is −0.185 +/− 0.018 kcal/mol with a maximum binding energy for the lowest energy pose of −0.258 kcal/mol. Binding of ivabradine to the human HCN1 open pore appears largely driven by van der Waals and hydrophobic interactions, with no consistent hydrogen bonding, anion-π or π-stacking interactions observed. Notably, no part of the ivabradine molecule fits into the hydrophobic groove generated by residues C358, Y386, and A387. Consistent with this observation, it was previously shown that the equivalent mutation to A387V in HCN4 channels (A507V) did not affect ivabradine block of HCN channels^64^. Interactions with these residues are weaker and less specific than for ligands such as clonidine, alinidine and lidocaine. Similar results were observed for zatebradine and cilobradine (Supp. Figs 5 and 6). Our findings are consistent with the model that ivabradine requires an open channel to access the binding site, and that outward current favours blocker binding. Our data also indicates that the “ligand trapped” closed conformation is not the same as the apo-closed conformation observed in the cryo-EM structure, since the apo-closed pore cavity lacks the volume necessary to accommodate any of the inhibitors we examined.

## Discussion

Targeted inhibition of HCN channels has strong therapeutic potential as anti-convulsants, analgesics, anti-depressants, anti-psychotics, and bradycardic agents without adverse effects on pulmonary and vascular smooth muscle tone. However, the lack of channel or isoform specificity and low affinity for most currently available inhibitors hinders their use. Thus, to aid in the development of higher affinity molecules, a detailed understanding of drug-channel interactions are required. We therefore sought to investigate molecular details of block of 9 known inhibitors of HCN channels (with differing scaffolds) and identify residues involved in their binding.

### Drug binding in the central cavity

The first intriguing result obtained from our docking experiments is that for each of the ligands examined, 0 of 500 docks to the closed human HCN1 pore contained ligands bound in the cavity. Our estimates using the CHARMM-GUI webserver suggest that the cavity can accommodate only 2–5 waters in this space with a diameter measured at Y386 of opposite subunits of 7.7 Å. Given that ligands such as ivabradine and ZD7288 are much larger and have been suggested to bind in the open state and be trapped in the closed state^57,65^, our data suggests that the ligand-trapped closed state differs from the apo-closed state that was observed in the cryo-EM structure.

Docking of these ligands to the open pore model of human HCN1 was more successful, with 100% of docking poses for each drug residing in the pore cavity. The residues that appear to be most critical for ligand binding of these pore blockers are C358, A383, Y386, A387, V390. Residues C358, Y386, and A387 appear to generate a hydrophobic groove that helps to conformationally restrict the ligands such as clonidine and alinidine, whose rings can fit. Binding of these ligands appears to be driven by a hydrogen bonds, anion-π and π-stacking interactions within this hydrophobic groove. On the other hand, “bradine” inhibitors appear to bind to the HCN1 open pore in a geometrically unconstrained manner via primarily van der Waals and hydrophobic interactions, and are sterically hindered from similar coordinated binding to the hydrophobic groove used for clonidine and alinidine binding. This lack of strong interactions and high rotational freedom within the pore cavity may provide a molecular basis for the low (2–10 µM) affinity of “bradines” to HCN channels. This may present a region that may be exploited for the development of higher affinity pore-blocking ligands. This idea is supported by the examination of binding of ZD7288 and “caine” inhibitors (such as lidocaine). The “caine” inhibitors can form parallel displaced pi interactions with Y386 and fit sterically into the pore groove, and thus dock in a relatively small number of clusters (≤5; Fig. 4). ZD7288 can form H-bond interactions, however, does not have a portion that can fit within this pore groove and form π-stacking interactions, and thus is relatively unconstrained in its binding (Fig. 5). Unfortunately, this lack of conformational restriction in the pore for ligands such as ZD7288 and ivabradine may present serious challenges for solving ligand bound structures using x-ray crystallography or cryo-EM approaches.

### Comparison with Previous Studies

Previous studies have attempted to examine the binding of ivabradine and ZD7288 to HCN channels using computational docking approaches^51,64,84,85^. Unfortunately, these studies required the use of homology model generated using prokaryotic KcsA^51,64,85^ and MthK^84,85^ channel as templates for the closed and open conformations, as an atomic resolution structure of HCNs or more closely related channels were not yet available at the time. Our study extracted the closed pore-domain from the the high-resolution structure of human HCN1 generated by cryo-EM experiments (PDB: 5U6O) to dock against. Structural alignment of the closed model derived from KcsA (Fig. 1A; red) and the closed pore extracted from the cryo-EM structure (Fig. 1A; cyan) indicate that there is a large difference between the structures. Firstly, the pore cavity in our models are lined by S6 residues C358, A383, Y386, A387, V390, G391, T394, A395, and Q398 in the closed and open states. This is largely in agreement with previous reports^50,51,64^, however differs for 2 key residues. L357 and F389 do not line the pore cavity. In the cryo-EM structure, the sidechain of F389 is faces S5 residues I298, I302, and M304 of the same chain and T384 of the neighbouring S6. This seems more reasonable than previously suggested, since the energetic cost of hydrating a phenylalanine group within the pore would be greater than if the residue was buried amongst non-polar residues. Similarly, in the cryo-EM structure, L357 packs as part of the interface between the pore-helix of one subunit and the N-terminal region of the neighbouring S6. These differences may play an important role in drug binding models, since many of the HCN inhibitors (such as bradines and ZD7288) largely interact with the channel through hydrophobic interactions and van der Waals forces, rather than through electrostatic interactions and hydrogen bonds. Most notably, the cryo-EM structure differs from previous models in the position of residue Y386 relative to the pore helix (Supp. Fig. 3). The distance between C358 and Y386 is 9.2 Å in the KcsA derived model, versus only 4.8 Å in the high-resolution structure. It is this difference that enables ivabradine to bind above residue Y386 in previous studies^64^, while it is sterically restricted from this space in our study. This difference also has a carry-over effect on the generation of an open model. Our open pore model was derived using the open pore observed in the cryo-EM structure of the closely related CNG channel TAX-4^63^. In the KcsA-based model of HCN4, I510 (equivalent to residue 390 in human HCN1) does not interact with ivabradine directly, but was proposed to stabilize the conformation of Y506 (equivalent to Y386 in HCN1) which does interact with the ligand^64^. However, our model, derived from the atomic resolution structures of HCN1 and TAX-4 CNG channels, indicates that ivabradine and ZD7288 bind below Y386 and can interact directly with V390 of HCN1 channels (Figs 5 and 6).

## Conclusions

Overall, our study provide novel insights into the mode of binding for 9 known inhibitors of HCN channels, and indicate that the inhibitor bound-closed pore state of HCN1 must differ from the apo-closed pore state observed in the high resolution structure. We also identify a hydrophobic groove within the pore cavity lined by residues C358, Y386, and A387 that appears to conformationally restrict ligands and has the potential to be exploited for the development of higher affinity molecules. Our results also help explain the molecular basis of the low-affinity binding of these inhibitors.

## Methods

### Model Building and Docking Calculations

The closed pore domain (S5-P-loop-S6) of human HCN1 (residues 296–402) was extracted from the PDB (5U6O or 5U6P)^62^ and processed through the CHARMM-GUI PDB Reader^86^ which enabled the corrections of any missing atoms and/or side-chains. In the absence of the crystal structure of an human HCN1 channel with an open pore domain, we turned to homology modelling using the template of the eukaryotic CNG channel, TAX-4 (PDB 5H3O)^63^. Sequences of the S5-pore-S6 residues were aligned using ClustalΩ^87^ (Supp. Fig. 1) and used to generate a homology model using ICM-Pro^88^ (Molsoft LLC, LaJolla). The model was processed through CHARMM-GUI PDB Reader^86^ to assist with repairing any missing atoms, repair any improper bond lengths or angles, and provide the appropriate PDB format for used for docking.

Preparation of the ligands and HCN1 channel pore domains in the open or closed states for docking experiments was performed using AutodockTools4 (ADT4)^89^. Grid parameter files and grid maps were generated by AutoGrid 4.2 within ADT with a grid spacing of 0.375 Å and positioned to exclude extracellular binding of the ligands. Each ligand was independently docked 500 times using Lamarckian GA docking algorithm^90^ with the maximum number of energy evaluations set to 2 500 000. Docking was performed on single processors of the supercomputer cluster Briaree (Compute Canada/CalculQuébec). Prior knowledge that these ligands are pore blockers enabled us to remove any docked poses outside of the pore cavity.

### Clustering Analysis

To account for the four-fold symmetry of the HCN pore, docked ligand poses were rotated into the same quadrant. Following this, ligands were clustered by (i) position in the pore and (ii) the orientation of functional groups. This was achieved by simplifying each ligand to a vector generated from the centroid of atoms selected in different regions of the molecule, as demonstrated for ivabradine (Supp. Fig. 1A). Table 1 lists the atoms used to determine each of the centroids determined for each ligand.

**Table 1:** Simplified ligand vectors used for clustering were generated by calculating 3–4 centroids within each ligand. The atoms used to calculate each centroid is listed.

Ligands	Centroid 1	Centroid 2	Centroid 3	Centroid 4
Clonidine	C7, C8, & C9	N1	C1 & C4	
Alinidine	C4 & C7	N1	C10, C11, & C12	
Lidocaine	C8 & C11	C6	N2	
Mepivacaine	N2	C13 & C10	N1	C6 & C3
Bupivacaine	N2	C5 & C8	N1	C12 & C15
ZD7288	C3 & C6	C9 & N2	N4	
Ivabradine	C20 & C21	C2	C7 & C12	
Zatebradine	C19 & C22	C2	C7 & C12	
Cilobradine	C3 & C6	C11 & C14	C19 & C24	

To cluster, using our simplified molecules, we generated a variant of Silhouette clustering^91^ to which we added a scoring function in order to find a suitable number of clusters without user bias. A “reference dock” is selected at random to generate the first cluster. The distance of every other dock is then compared to this reference position via the sum of linear distance between each of the centroids from our selections in Table 1 according to:

$${\rm{D}}=\sum _{i=1}^{n}{a}_{i}-{b}_{i}$$

where D is the sum of the distances between each centroid, n the number of centroids we selected as best representative of the whole molecule, and a and b represent the docks being are compared.

If D lies within a defined threshold distance it is merged to the cluster, otherwise a new cluster is created. 4 Å (equivalent to a weak hydrogen bond) was selected as the starting threshold.

Since the number of clusters calculated is dependent on the threshold set, we added a scoring function (S) to each cluster to choose the optimal number.

$$S=\frac{Inside\,Distance}{Outside\,Distance}$$

with Inside Distance defined as:

$$I=Max\,{(\sum _{i=1}^{n}{x}_{i}-c)}_{i}$$

With c being the cluster’s global centroid and x the centroid for each individual dock inside the cluster.

The Outside Distance is defined as:

$$O=Max{(\sum _{i=1}^{n}{y}_{i-}c)}_{i}$$

with y being the centroid for every dock from the nearest cluster.

Ideally, S should be <1 for all clusters calculated, which indicates every cluster is justified and the number of clusters is well determined. If the S determined for any cluster >1, the threshold is automatically lowered by 0.1 Å for each iteration until every S falls below 1. If S remains >1 for every iteration until 0, the algorithm selects for the threshold yielding the lowest maximum S and the number of clusters is determined accordingly.

## Supplementary information

Supplemental Figures

## Data Availability

The datasets generated during and/or analysed during the current study are available from the corresponding author on reasonable request.
