# Supplementary material for: Characterization of drug binding within the HCN1 channel pore
Source: Sci Rep. 2019 Jan 24;9:465. doi: 10.1038/s41598-018-37116-2 (PMC6345760; doi:10.1038/s41598-018-37116-2)
Supplement: Supplementary file 1 — Supplemental Figures [file 41598_2018_37116_MOESM1_ESM.pdf]

Characterization of drug binding within the HCN1 channel pore.

Authors: Jérémie Tanguay[1], Karen M. Callahan[2], Nazzareno D'Avanzo[2]

Affiliations:

[1] Department of Physics, Université de Montréal, Montréal, Canada.

[2] Department of Pharmacology and Physiology, Université de Montréal, Montréal, Canada.

For correspondence: Ph: (514) 343-5634, FAX: (514) 343-7146,
e-mail: nazzareno.d.avanzo@umontreal.ca

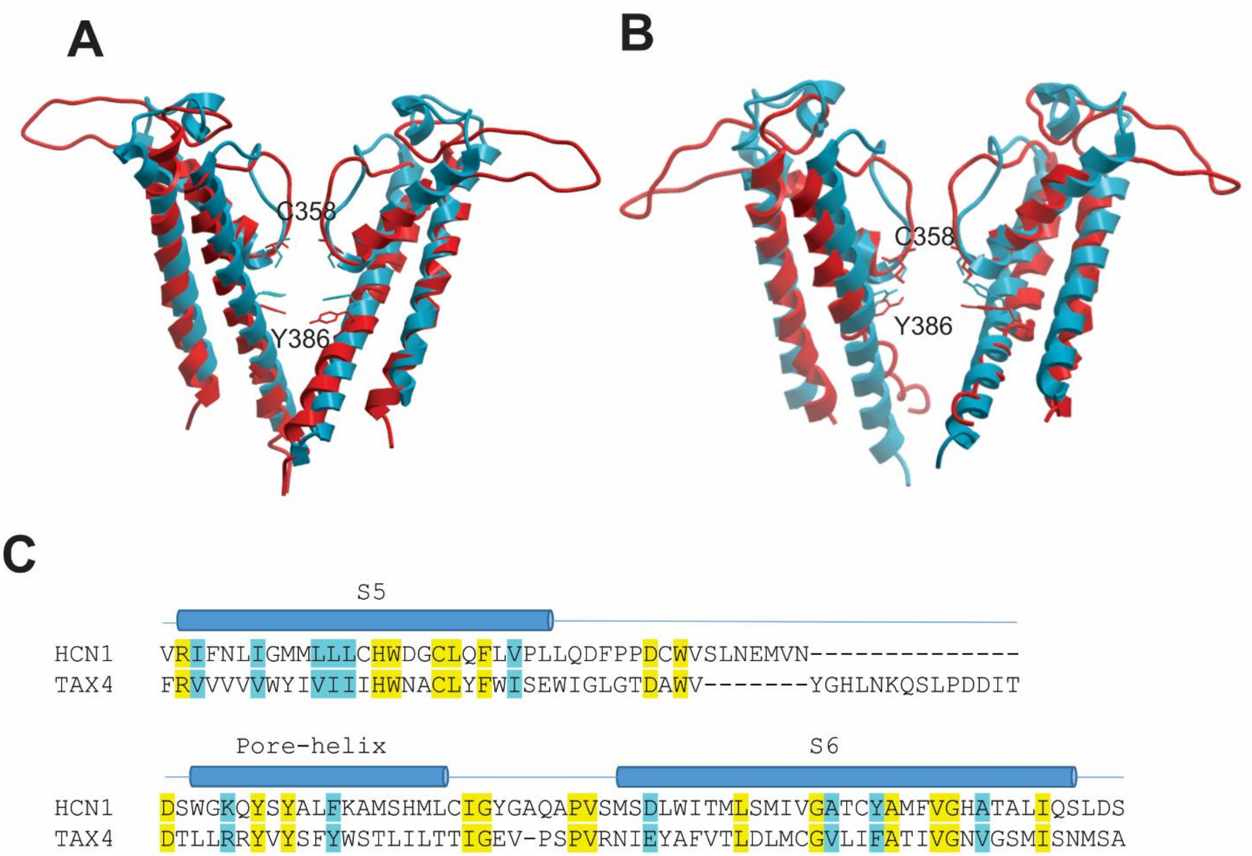

Supp. Fig. 1. HCN1 Homology Modeling. (A) Closed state pore models either derived from KcsA (red) or taken from the high resolution cryo-EM structure (blue) (PDB: 5U6O). **(B)** Open state pore models either derived from KcsA (red) or using the more closely related eukaryotic CNG channel, TAX-4 (PDB 5H3O) as a template (blue). Notably, the distance between C358 and Y386 is greater in the KcsA based model in both the open and closed states, compared to the model derived from the cryo-EM structures. **(C)** Sequence alignment of the pore domains of human HCN1 and the eukaryotic TAX-4 used for generating the open state homology model.

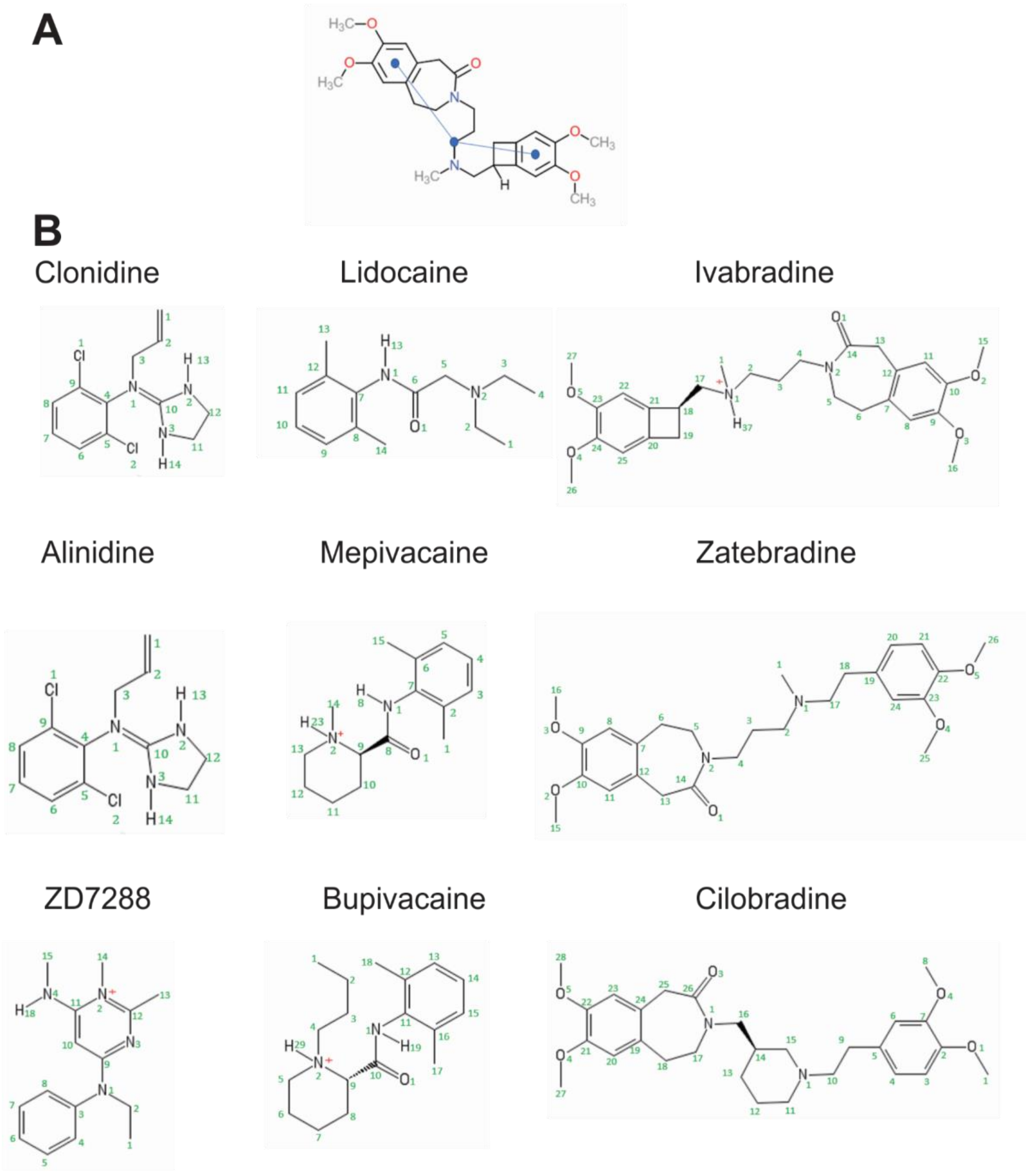

Supp. Fig. 2. HCN inhibitors used for docking. (A) Docking poses were clustered using a simplified vector model, whereby, calculated from the the centroids (dots) of 3 – 4 functional groups on the ligand were calculated for each ligand. Atoms used to calculate centroids are shown in Table 1. **(B)** Chemical structures of the inhibitors used. Atom numbers are labelled in light green.

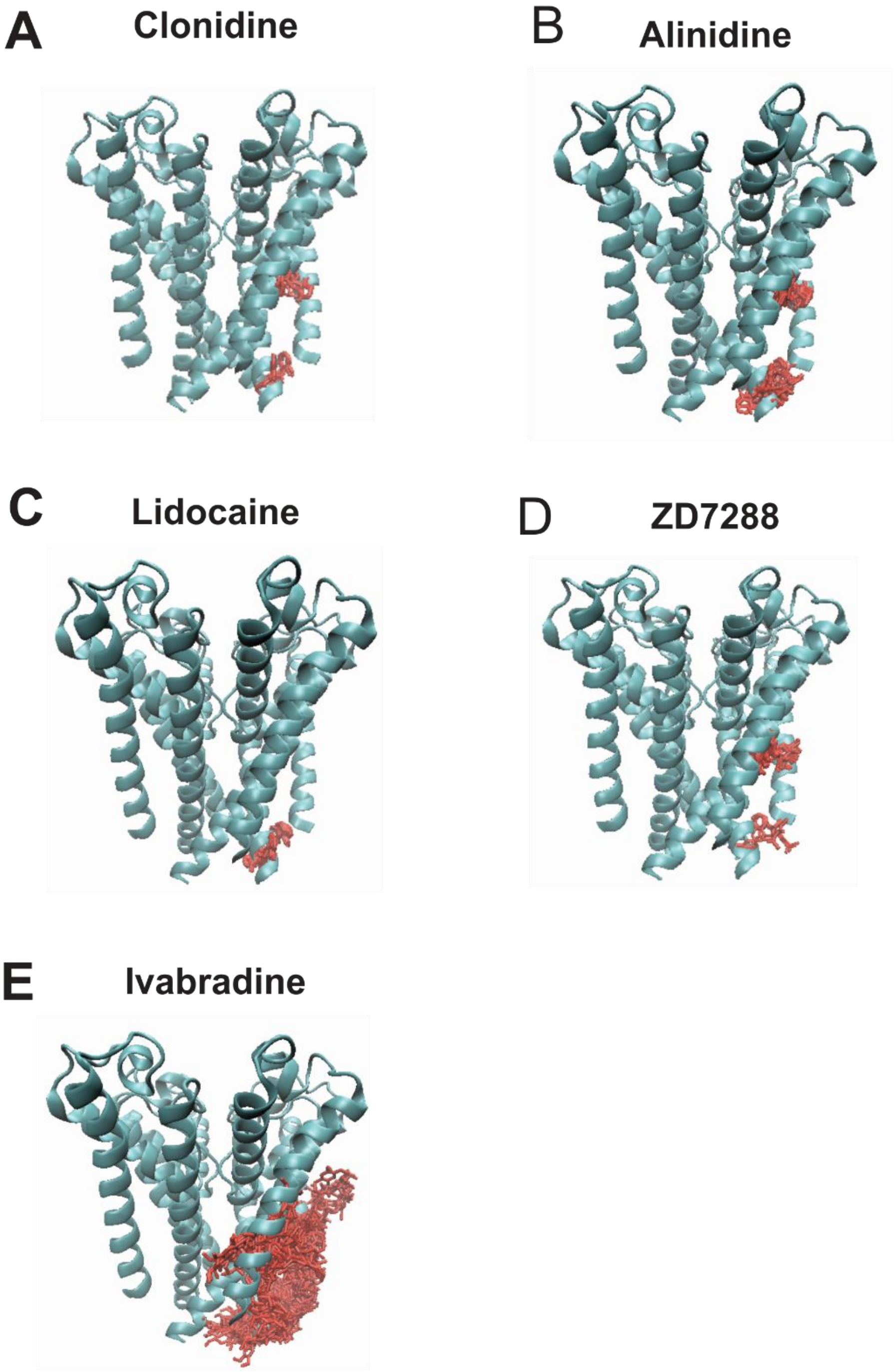

Supp. Fig. 3. **Docking of HCN blockers to the cAMP-bound closed HCN1 pore.** The results from 500 attempts to dock clonidine **(A)** alinidine **(B)** lidocaine **(C)** ZD7288 **(D)** and ivabradine **(E)** to the closed pore of the cryo-EM structure (PDB: 5U6O). Despite these ligands being known to block or be "trapped" in the closed state, none of the 500 docked poses for any of these inhibitors were observed in the pore cavity. These data indicate that the cAMP-bound closed pore conformation must differ from the ligand-trapped closed pore.

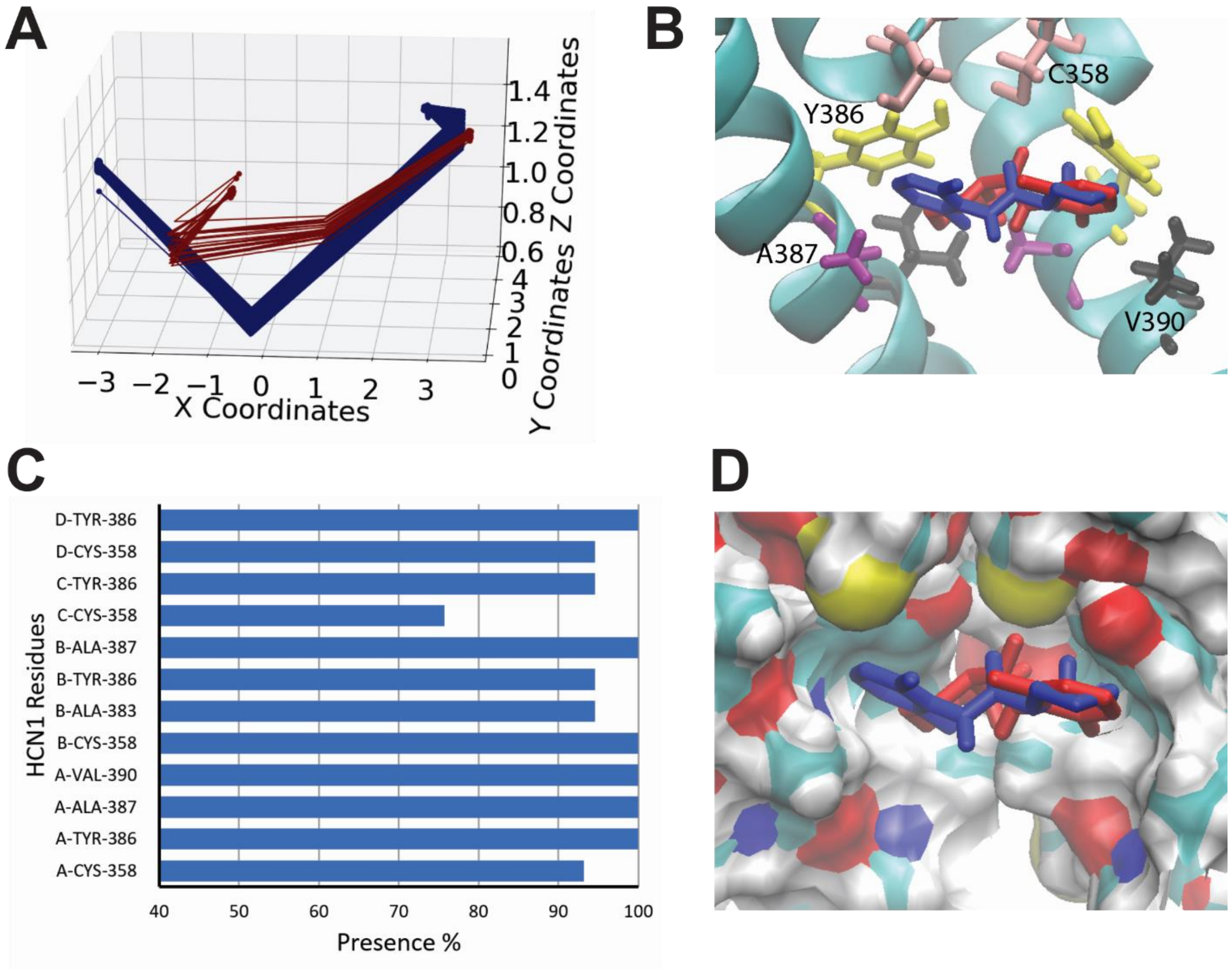

Supp. Fig. 4. **Docking of mepivacaine to the open HCN1 pore. (A)** Clustering analysis of the 500 docking attempts. Docks can be placed into 2 clusters according to our automated algorithm. **(B)** The representative poses from cluster 1 (blue) and cluster 2 (red) are shown. C358 (pink), Y386 (yellow), A387 (purple) and V390 (black) are also highlighted **(C)** The frequency of residues within 4Å of the ligand for each pose was assessed and is indicated as a percentage. **(D)** Both docking conformations allow for the the mepivacaine rings to be oriented into the hydrophobic groove in the pore cavity.

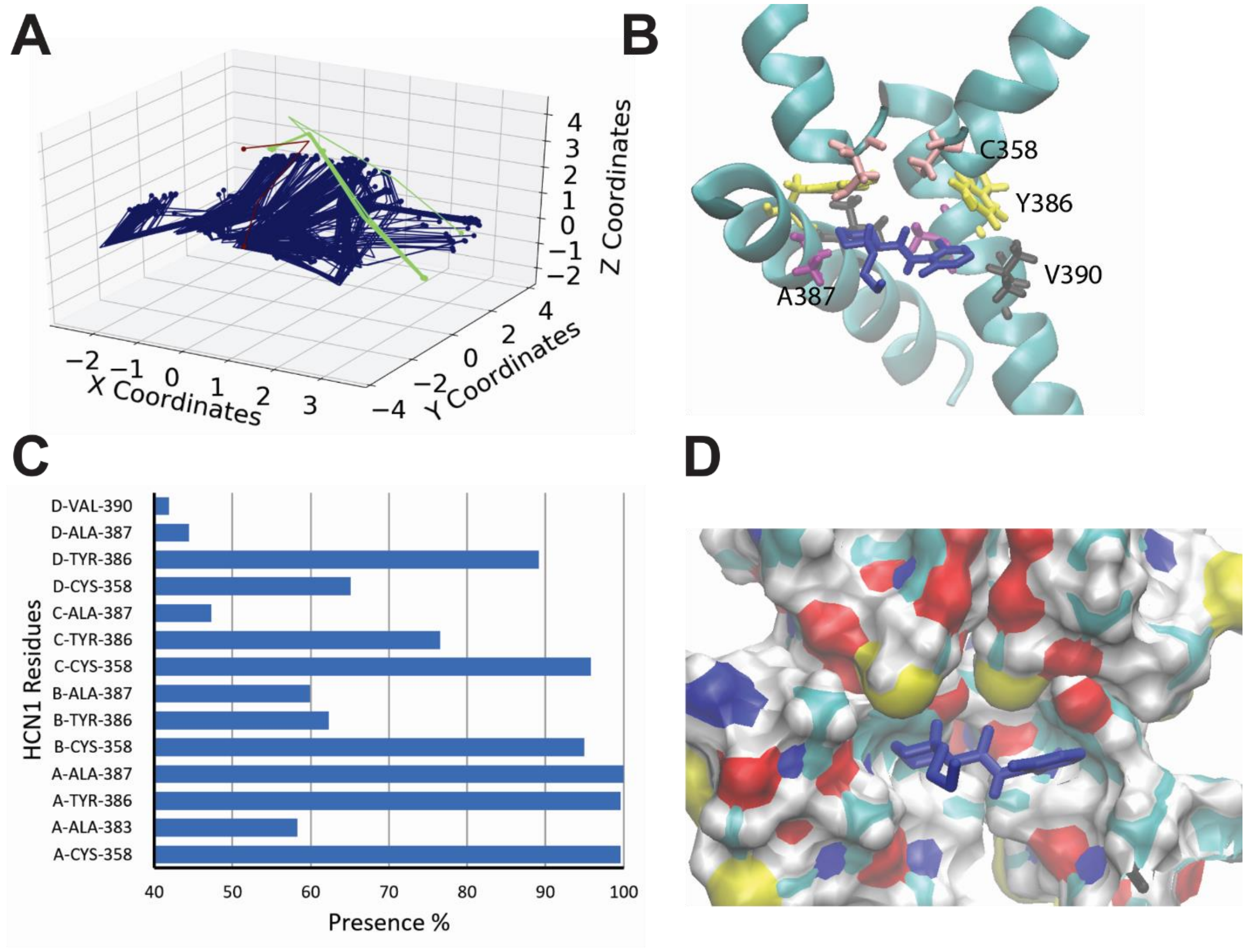

Supp. Fig. 5. Docking of bupivacaine to the open HCN1 pore. (A) Clustering analysis of the 500 docking attempts. Docks can be placed into 3 clusters according to our automated algorithm. **(B)** The representative poses from cluster 1 (blue) which contains the majority of docks is shown with residues C358 (pink), Y386 (yellow), A387 (purple) and V390 (black) highlighted **(C)** The frequency of residues within 4Å of the ligand for each pose was assessed and is indicated as a percentage. **(D)** Bupivacaine docks similarly to mepivacaine, with both rings to be oriented into the hydrophobic groove in the pore cavity lined by by residues C358, Y386, and A387, however, the extended butyl group on the aromatic ring points toward the pore axis, which we predict would better occlude permeating ions.

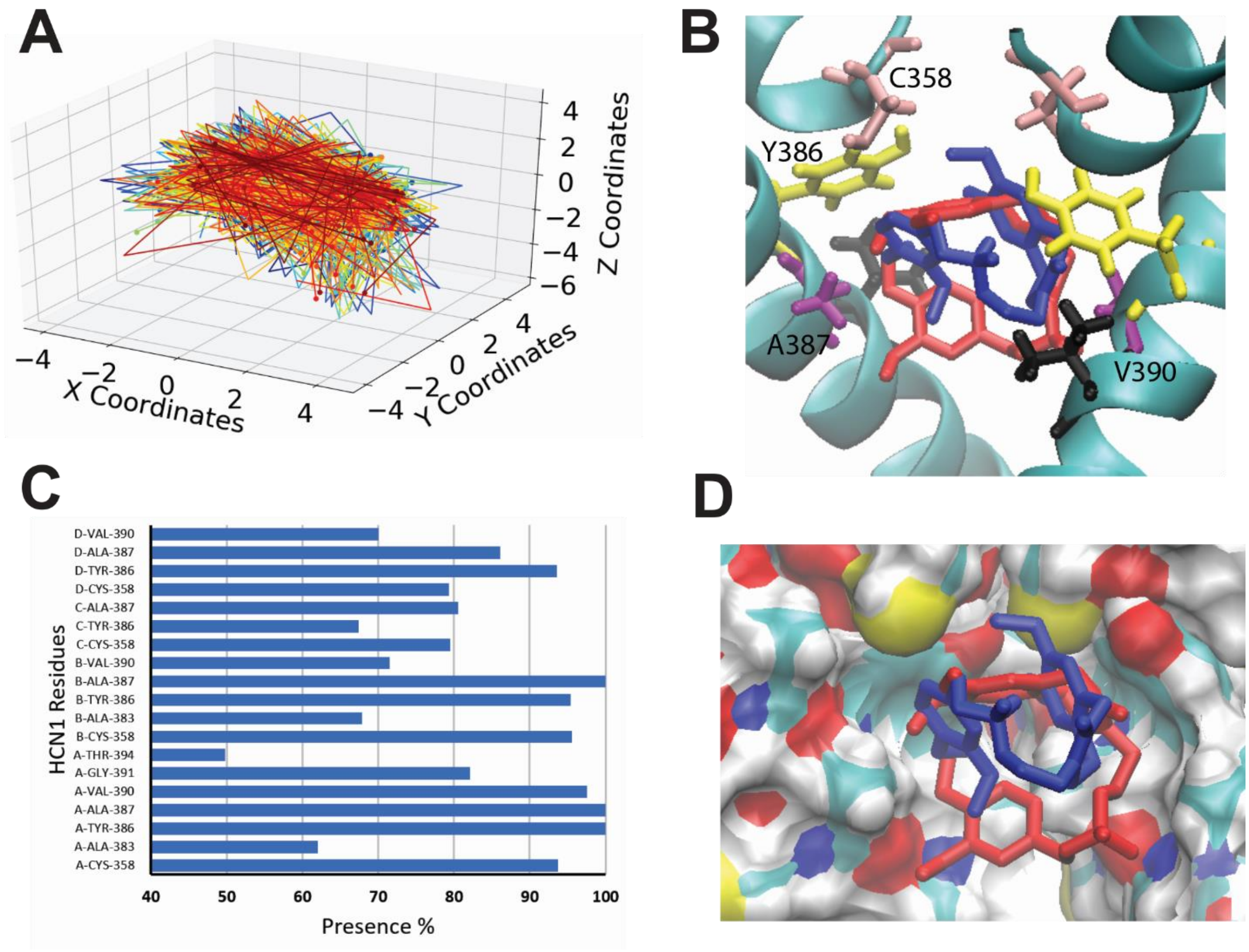

Supp. Fig. 6. Docking of zatebradine to the open HCN1 pore. (A) Clustering analysis of the 500 docking attempts. Docks cannot be placed into clusters according to our algorithm with the ligand freely rotating 360° around the pore axis. **B)** Similar to the two docking poses shown, zatebradine takes on a U-shape in the pore in all docking formations. Residues C358 (pink), Y386 (yellow), A387 (purple) and V390 (black) are highlighted. **(C)** The frequency of residues within 4Å of the ligand for each pose was assessed and is indicated as a percentage. **(D)** Similar to ivabradine, no portion of zatebradine can fit into the hydrophobic groove within the pore cavity.

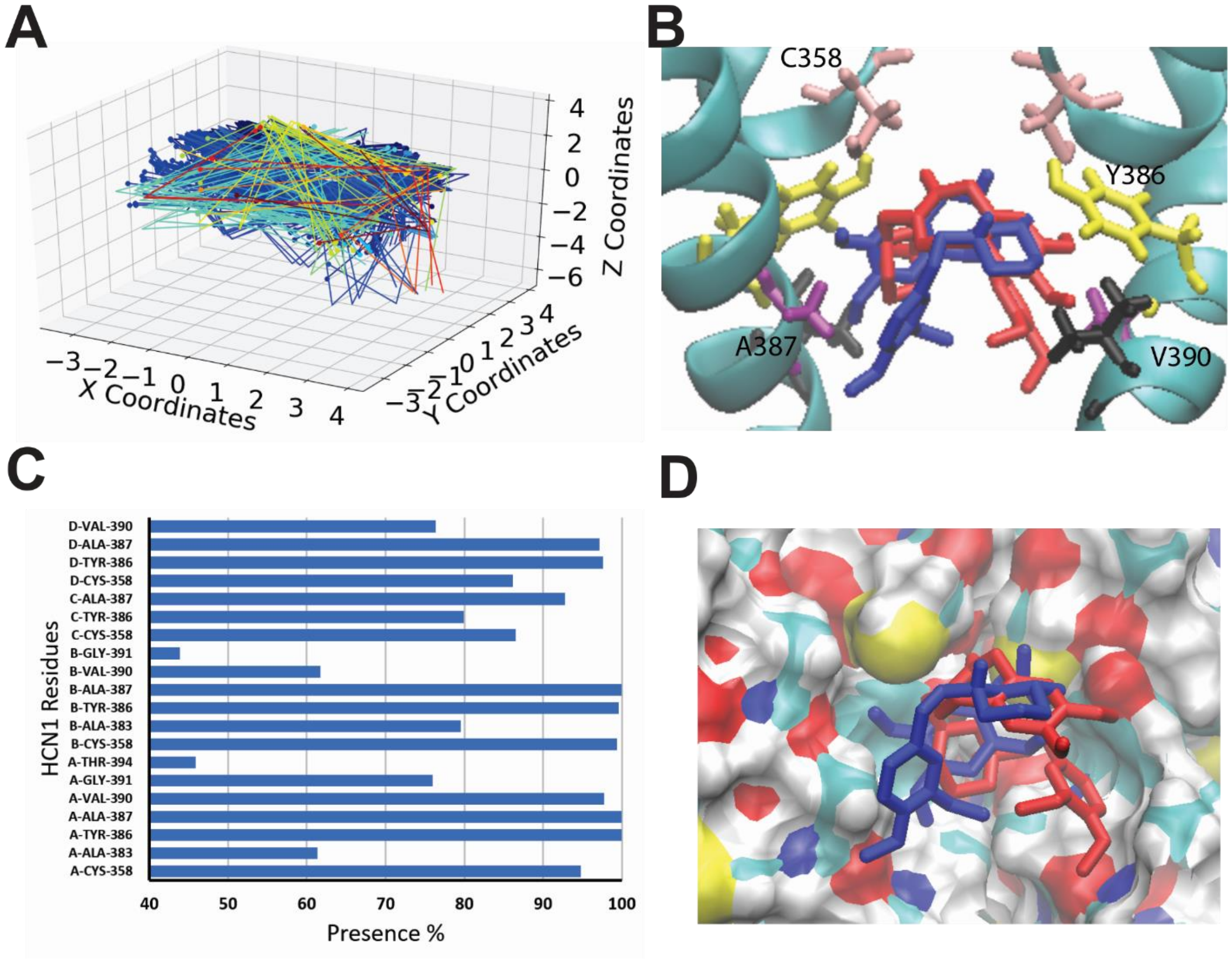

Supp. Fig. 7. Docking of cilobradine to the open HCN1 pore. (A) Clustering analysis of the 500 docking attempts. Docks cannot be placed into clusters according to our algorithm with the ligand freely rotating 360° around the pore axis. **(B)** Similar to the two docking poses shown, cilobradine takes on a U-shape in the pore in all docking formations. Residues C358 (pink), Y386 (yellow), A387 (purple) and V390 (black) are highlighted. **(C)** The frequency of residues within 4Å of the ligand for each pose was assessed and is indicated as a percentage. **(D)** Similar to ivabradine and zatebradine, no portion of zatebradine can fit into the hydrophobic groove within the pore cavity.
